# Glucansucrase Gtf180-ΔN of *Lactobacillus reuteri* 180: enzyme and reaction engineering for improved glycosylation of non-carbohydrate molecules

**DOI:** 10.1007/s00253-016-7476-x

**Published:** 2016-04-06

**Authors:** Tim Devlamynck, Evelien M. te Poele, Xiangfeng Meng, Sander S. van Leeuwen, Lubbert Dijkhuizen

**Affiliations:** 1 Microbial Physiology, Groningen Biomolecular Sciences and Biotechnology Institute (GBB), University of Groningen, Nijenborgh 7, 9747 AG Groningen, The Netherlands; 2 Centre for Industrial Biotechnology and Biocatalysis, Department of Biochemical and Microbial Technology, Faculty of Bioscience Engineering, Ghent University, Coupure Links 653, 9000 Ghent, Belgium

**Keywords:** Glycosylation, Glucansucrase, Catechol, *Lactobacillus reuteri*, Acceptor reaction, Enzyme engineering

## Abstract

Glucansucrases have a broad acceptor substrate specificity and receive increased attention as biocatalysts for the glycosylation of small non-carbohydrate molecules using sucrose as donor substrate. However, the main glucansucrase-catalyzed reaction results in synthesis of α-glucan polysaccharides from sucrose, and this strongly impedes the efficient glycosylation of non-carbohydrate molecules and complicates downstream processing of glucosylated products. This paper reports that suppressing α-glucan synthesis by mutational engineering of the Gtf180-ΔN enzyme of *Lactobacillus reuteri* 180 results in the construction of more efficient glycosylation biocatalysts. Gtf180-ΔN mutants (L938F, L981A, and N1029M) with an impaired α-glucan synthesis displayed a substantial increase in monoglycosylation yields for several phenolic and alcoholic compounds. Kinetic analysis revealed that these mutants possess a higher affinity for the model acceptor substrate catechol but a lower affinity for its mono-α-d-glucoside product, explaining the improved monoglycosylation yields. Analysis of the available high resolution 3D crystal structure of the Gtf180-ΔN protein provided a clear understanding of how mutagenesis of residues L938, L981, and N1029 impaired α-glucan synthesis, thus yielding mutants with an improved glycosylation potential.

**Electronic supplementary material:**

The online version of this article (doi:10.1007/s00253-016-7476-x) contains supplementary material, which is available to authorized users.

## Introduction

Glycosylation is a versatile tool to enhance the physicochemical and biological properties of small non-carbohydrate molecules (Desmet et al. 2012). This may result in an increased solubility of hydrophobic compounds (Torres et al. 2011) and an improved stability of labile molecules against light and oxidation (Yamamoto et al. 1990). Furthermore, glycosylating medium- and long-chain alcohols yields alkyl glycosides or alkyl polyglycosides, a class of eco-friendly and non-ionic surfactants displaying a high surface activity and good biodegradability (Ojha et al. 2013).

The chemical synthesis of glycosides requires the use of toxic catalysts and involves many protection and deprotection steps, resulting in low overall yields. Biocatalysis offers an alternative method circumventing multistep synthesis and generating 5-fold less waste (de Roode et al. 2003). In nature, glycosylation is catalyzed by Leloir glycosyltransferase enzymes (EC 2.4.-.-), using nucleotide-activated sugars as donor substrates. Despite their high efficiency and specificity, the breakthrough as glycosylation catalysts is hampered by the high price of their donor substrates (Lairson et al. 2008). Glycosidases (EC 3.2.-.-) in turn suffer from low yields when applied in the synthetic direction (Desmet and Soetaert 2012).

Glycoside hydrolase enzymes such as glucansucrases (GS) provide an excellent alternative for enzymatic glycoside synthesis. These enzymes belong to glycoside hydrolase family 70 (GH70) (Lombard et al. 2014) and catalyze the conversion of the cheap donor substrate sucrose into α-glucan polysaccharides, thereby linking the α-d-glucopyranosyl units by (α1 → 2), (α1 → 3), (α1 → 4), or (α1 → 6) bonds, depending on the enzyme specificity (Monchois et al. 1999; van Hijum et al. 2006). Moreover, GS are promiscuous towards a wide range of acceptor substrates (Leemhuis et al. 2013; Monsan et al. 2010). They can use saccharides such as maltose as acceptor substrate to catalyze the synthesis of various oligosaccharides (Yoon and Robyt 2002). Glycosylation of non-carbohydrate acceptor substrates, such as l-ascorbic acid (Kim et al. 2010) and luteolin (Bertrand et al. 2006), also has been reported. The usefulness of GS enzymes as a glycosylation biocatalyst is further demonstrated by a number of patent applications by Auriol et al. (2012), in which the synthesis of a wide array of phenolic compounds with *Leuconostoc* glucansucrases is claimed.

A remarkable characteristic shared by all GS is their ability to add multiple α-d-glucopyranosyl moieties to one acceptor substrate, forming α-d-glucosides of different sizes and structures. A prominent example concerns the glycosylation of acceptor substrates by the GtfA enzyme of *Lactobacillus reuteri* 121 (Kralj et al. 2004): after incubation with catechol and sucrose, several glycosylated catechol products up to DP5, differing in their combination of (α1 → 4) and (α1 → 6) linkages, were characterized (te Poele et al. 2016). From an industrial perspective, the synthesis of only one glycoside is desired in order to facilitate downstream processing. In addition to the production of a mixture of α-D-glucosides, glucansucrases also synthesize rather large amounts of α-glucan polysaccharides from sucrose under these conditions. This is in fact their main reaction but in this case an unwanted side reaction lowering the yield of the glycosylated acceptor substrates and complicating their downstream processing. In this paper, a combination of reaction- and enzyme engineering was applied to explore the potential of the N-terminally truncated glucansucrase Gtf180 from *L*. *reuteri* 180 (Gtf180-ΔN, retaining wild-type activity and specificity) (Pijning et al. 2008) as a glycosylation biocatalyst, aiming to suppress the competing α-glucan synthesis reaction as much as possible. Screening of a previously constructed mutant library, targeting 10 amino acid residues involved in the acceptor substrate binding subsites +1 and +2 (Meng et al. 2016; Meng et al. 2015), yielded mutants with an impaired α-glucan synthesis. As will be demonstrated, this substantially enhanced the conversion of a wide range of phenolic and alcoholic molecules into their α-D-glucosides, and also shifted the glycoside distribution pattern towards monoglycosylation.

## Materials and methods

### Production and purification of recombinant Gtf180-ΔN (mutants)

Recombinant, N-terminally truncated Gtf180-ΔN from *L*. *reuteri* 180 and derived mutant enzymes (Table S1) were produced and purified as described previously (Kralj et al. 2004; Meng et al. 2015).

### Glucansucrase activity assays

Enzyme activity assays were performed at 37 °C with 100 mM sucrose in 25 mM sodium acetate (pH 4.7) and 1 mM CaCl_2_ unless stated otherwise. Samples of 100 μL were taken every minute over a period of 8 min and immediately inactivated with 20 μL 1 M NaOH for 30 min. The released glucose and fructose were quantified enzymatically by monitoring the reduction of NADP with the hexokinase and glucose-6-phosphate dehydrogenase/phosphoglucose isomerase assay (Roche) as described previously (Van Geel-Schutten et al. 1999; Mayer 1987), allowing the determination of the total (fructose release) and hydrolytic (glucose release) activities, and calculation of the transglycosylation activity. The α-glucan synthesis potential (α-GSP) is defined as the ratio of transglycosylation activity over total activity.

One unit (U) of total activity corresponds to the release of 1 μmol fructose from 100 mM sucrose in 25 mM sodium acetate (pH 4.7) and 1 mM CaCl_2_ at 37 °C. For the comparison of different reaction conditions and mutants, 4 U/mL enzyme was added to the incubations, unless stated otherwise.

### Production and purification of glycoside products

The glycosylation of catechol, resorcinol, hydroquinone, and butanol was carried out at 100 mL scale, by incubating 1 U/mL Gtf180-ΔN at 37 °C in 25 mM sodium acetate (pH 4.7) and 1 mM CaCl_2_ with 400 mM acceptor substrate and 1000 mM sucrose for 2 h. Alternatively, hexanol and octanol were glycosylated in a biphasic system consisting of 20 % alcohol, 25 mM sodium acetate (pH 4.7), 1 mM CaCl_2_, and 1000 mM sucrose, while stirring was achieved in a shaker at 100 rpm. The reactions were terminated by incubating the reaction mixture at 95 °C for 10 min. Most of the fermentable sugars were subsequently removed by fermentation with the yeast *Saccharomyces cerevisiae* (Fermentis Ethanol Red®) at pH 4.0 and 30 °C (De Winter et al. 2011). Twenty grams per liter peptone and 10 g/L yeast extract were added to support growth. After 24 h incubation, the yeast cells were removed by centrifugation (10,000×*g*, 4 °C, 10 min) after which the supernatant was concentrated by evaporating in vacuo. The glycoside products were subsequently purified from the residue by column chromatography using silica gel (pore size 60 Å, particle size 230–400 mesh) as the stationary phase. The eluent consisted of ethyl acetate-methanol-water (30:5:4 by volume) in case monoglucosides were purified and ethyl acetate-methanol-water (30:6:4 by volume) for the purification of diglucosides.

### HPLC analysis

HPLC analysis of phenolic acceptor molecules and their α-d-glucosides was performed on an Adsorbil amine column (250 × 4.6 mm, 10 μm) with acetonitrile (solvent A) and 50 mM ammonium formate (pH 4.4, solvent B) as the mobile phase. The flow rate and temperature were set at 1.0 mL/min and 35 °C, respectively. The following gradient elution was used: 95 % of solvent A (0–5 min), 5–40 % solvent B (5–22 min), 80 % solvent B (22–25 min), and again 95 % of solvent A (25–29 min). Detection of the phenolic acceptor substrates and their α-d-glucosides was achieved with an UV detector (276 nm). Before being subjected to HPLC analysis, the samples were diluted 200 times in 80 % methanol. Calibration of the obtained peaks was accomplished using standard curves of the purified glycosides. All HPLC analyses were performed in duplicate.

### TLC analysis

TLC analysis was performed on silica gel 60 F_254_ plates (Merck). The eluent consisted of ethyl acetate-methanol-water (30:5:4 by volume). Detection was achieved by UV absorption (254 nm) and/or staining with 10 % (*v*/*v*) H_2_SO_4_ containing 2 g/L orcinol. The concentration of the alkyl α-d-glucosides was determined by scanning the charred plates with a ChemiDoc™ MP imaging system and subsequently analyzing the spots with Image Lab 4.0 software. Calibration of the obtained spots was accomplished using standard curves of the purified alkyl α-d-glucosides. There was a linear response in the range of 1–10 mM alkyl glucoside (determined experimentally). All TLC analyses were performed in triplicate.

### Kinetic analysis of Gtf180-ΔN (mutants)

Kinetic analysis of the Gtf180-ΔN (mutants) was based upon the method described by Dirks-Hofmeister et al. (2015) for the glycosylation of resveratrol with sucrose phophorylase. Kinetic parameters (*K*_m_ and *k*_cat_ values) for the acceptor substrates catechol and the mono-α-d-glucoside of catechol (catechol-G1), purified as described above, were determined using 10 different catechol (−G1) concentrations (ranging from 6.25 to 400 mM), while the concentration of the donor substrate sucrose had a constant value of 1000 mM. One unit per milliliter of Gtf180-ΔN (mutants) was added. Four samples were taken over a period of 3 min and immediately inactivated by incubating for 10 min at 95 °C. All samples were subjected to TLC analysis as described above. The charred plates were scanned with a ChemiDoc™ MP imaging system allowing analysis of the spots with Image Lab 4.0 software. Calibration of the obtained spots was accomplished using standard curves of the purified catechol-G1. Kinetic parameters were calculated by non-linear regression of the Michaelis-Menten equation with SigmaPlot v12.0.

### Structural characterization of purified α-d-glucosides

The structures of the purified α-d-glucosides were elucidated by a combination of 1D NMR (^1^H NMR and ^13^C NMR) and 2D NMR spectroscopy. Samples were exchanged twice in 300 μL D_2_O 99.9%_atom_ (Cambridge Isotope Laboratories, Andover, MA) with intermediate lyophilization. Finally, samples were dissolved in 650 μL D_2_O, containing acetone as internal standard (δ^1^H 2.225; δ^13^C 31.08). ^1^H NMR spectra, including ^1^H–^1^H and ^13^C–^1^H correlation spectra were recorded at a probe temperature of 298 K on a Varian Inova 600 spectrometer (NMR Department, University of Groningen, The Netherlands). 1D 600-MHz ^1^H NMR spectra were recorded with 5000 Hz spectral width at 16k complex data points, using a WET1D pulse to suppress the HOD signal. 2D ^1^H–^1^H COSY spectra were recorded in 256 increments in 4000 complex data points with a spectral width of 5000 Hz. 2D ^1^H–^1^H TOCSY spectra were recorded with MLEV17 mixing sequences with 50, 90, and 150 ms spin-lock times. 2D ^13^C–^1^H HSQC spectra were recorded with a spectral width of 5000 Hz in *t*_2_ and 10,000 Hz in *t*_1_ direction. 2D ^1^H–^1^H ROESY spectra with a mixing time of 300 ms were recorded in 128 increments of 4000 complex data points with a spectral width of 5000 Hz. All spectra were processed using MestReNov. 5.3 (Mestrelabs Research SL, Santiago de Compostela, Spain), using Whittaker Smoother baseline correction.

## Results

Glucansucrases prefer non-carbohydrate acceptor substrates with two vicinal hydroxyl groups (Bertrand et al. 2006), such as catechol. The latter has a high water solubility at room temperature, rendering the addition of co-solvents unnecessary. Glycosylation of catechol with the N-terminally truncated glucansucrase of *L*. *reuteri* 180 (Gtf180-ΔN) (Pijning et al. 2008) was chosen as the model reaction. Firstly, the reaction conditions were optimized towards maximal monoglycosylation and minimal α-glucan synthesis. Subsequently, the mutant library was screened, applying these optimal reaction conditions. Finally, the optimal reaction conditions identified for catechol glycosylation were also tested for glycosylation of other acceptor substrates.

### Reaction engineering of catechol glycosylation by wild-type Gtf180-ΔN

**Fig. 1:**
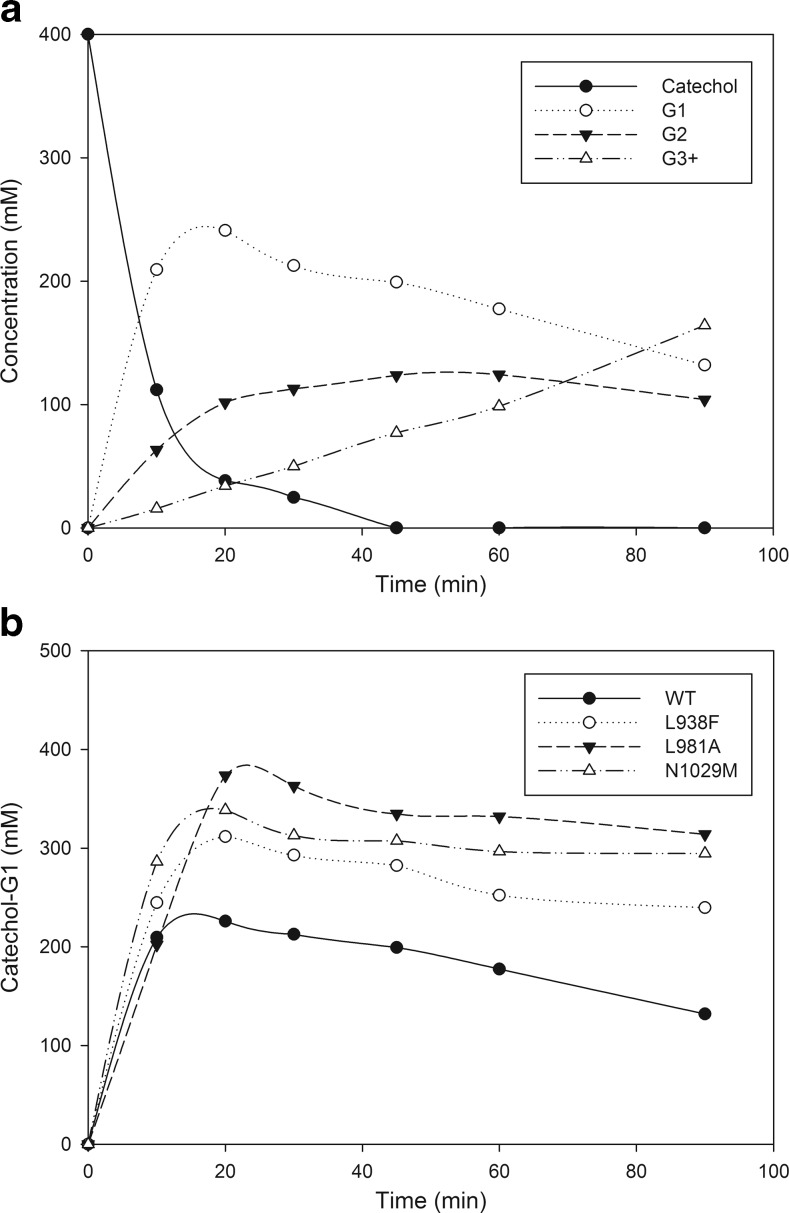
**a** Time-course synthesis of α-D-glucosides of catechol by WT Gtf180-ΔN (400 mM catechol, 1000 mM sucrose, 4 U/mL Gtf180-ΔN). *T* = 37 °C, pH = 4.7. **b** Time-course synthesis of catechol-G1 by WT Gtf180-ΔN and mutants derived (400 mM catechol, 1000 mM sucrose, 4 U/mL Gtf180-ΔN). *T* = 37 °C, pH = 4.7

The catechol acceptor concentration was optimized towards maximal monoglycosylation and minimal α-glucan synthesis. As shown in Fig. 1a, formation of the monoglucoside of catechol (catechol-G1) is kinetically controlled. Incubation for 20 min was sufficient to reach maximal catechol-G1 production, coinciding with catechol depletion. Catechol-G1 was subsequently irreversibly converted into diglucoside (catechol-3′G2 and catechol-6′G2) and further (catechol-G3+). The donor substrate sucrose was not depleted yet (data not shown).

Glycosylation reactions catalyzed by glucansucrases suffer from low thermodynamic favorability as pointed out by Liang et al. (2016). The production of high catechol-G1 concentrations therefore requires an excess of donor substrate sucrose to drive the reaction. We observed that the latter also had a stabilizing effect on the enzyme, allowing addition of relatively high acceptor substrate concentrations which would otherwise be detrimental for the enzyme activity as described previously (Meulenbeld and Hartmans 2000). Therefore, the sucrose concentration was set at 1000 mM. Kinetic analysis revealed that Gtf180-ΔN follows Michaelis-Menten kinetics at catechol acceptor substrate concentrations between 6.25 and 400 mM (Figure S1). The *K*_m_ value of Gtf180-ΔN for catechol was 103.3 mM which illustrated the need for high acceptor substrate concentrations (Table 1). Therefore, the catechol concentration was varied from 100 to 1000 mM while the sucrose concentration was kept constant at 1000 mM (Table 2). At catechol concentrations higher than 600 mM no glycosylated product was formed due to severe inhibition of enzyme activity by catechol. A catechol concentration of 500 and 600 mM only allowed partial conversion of catechol with monoglycosylation yields of 17 and 7 %, respectively. Reaction mixtures containing 400 mM catechol or less, displayed complete conversion of this acceptor substrate into α-d-glucoside products. Increasing the acceptor concentration from 100 to 400 mM resulted in an improvement in monoglycosylation yield from 49 to 60 %, whereas the synthesis of triglucosylated products was reduced (Table 2). At higher catechol concentrations, there indeed is an increased chance that the enzyme glycosylates a new acceptor substrate rather than glycosylating catechol-G1. Consequently, 400 mM catechol was chosen as the optimal acceptor concentration for the production of monoglucosides.

**Table 1:** Kinetic parameters of WT Gtf180-ΔN and mutants derived for catechol (6.25–400 mM) and catechol-G1 (6.25–400 mM) as acceptor substrates (with 1000 mM sucrose as donor substrate), and α-GSP of Gtf180-ΔN WT and mutants for sucrose as both donor and acceptor substrate

Enzymes	Catechol			Catechol-G1			α-GSP
	*K*_m_ (mM)	*k*_cat_ (1/s)	*k*_cat_/*K*_m_ (1/s.mM)	*K*_m_ (mM)	*k*_cat_ (1/s)	*k*_cat_/*K*_m_ (1/s.mM)	
WT	103.3 ± 8.5	757.4 ± 12.4	7.4	88.8 ± 17.1	863.3 ± 39.3	10.2	0.556
L938F	85.5 ± 4.0	1872.5 ± 74.7	21.9	91.1 ± 7.3	576.2 ± 44.0	6.3	0.341
N1029M	58.9 ± 6.4	449.4 ± 4.9	7.7	146.9 ± 19.3	126.2 ± 11.0	0.9	0.192
L981A	11.0 ± 1.3	203.2 ± 8.1	18.7	177.4 ± 7.0	69.4 ± 2.4	0.4	0.049

*T* = 37 °C, pH = 4.7. α-GSP is defined as the ratio of the transglycosylation activity over the total activity (measured with 1000 mM sucrose only)

**Table 2:** Effects of acceptor substrate concentration on the glycosylation yields and glucoside distribution of WT Gtf180-ΔN for the model acceptor substrate catechol (1000 mM sucrose, 4 U/mL Gtf180-ΔN)

Catechol (mM)	Catechol glucoside (mM)				Catechol glucoside distribution (%)		
	G1	G2_α1 → 3_	G2_α1 → 6_	G3+	G1	G2	G3+
600	39.6	−	−	−	100	−	−
500	86.8	< 10.0	< 10.0	−	96	4	4
400	241.1	33.8	68.0	57.3	60	25	14
300	170.1	27.5	56.0	46.3	57	28	15
200	107.2	17.9	37.6	37.2	54	28	19
100	49.4	9.3	18.1	23.1	49	27	23

*T* = 37 °C, pH = 4.7. All data given at maximal catechol-G1 yield (20 min incubation)

The *K*_m_ value of Gtf180-ΔN for the catechol-G1 acceptor substrate was 88.8 mM, which is lower than the value for catechol (103.3 mM). The *k*_cat_ values were 863.3 and 757.4 1/s, respectively (Table 1). Hence, under these conditions, Gtf180-ΔN glycosylation of catechol-G1 into catechol-G2 and further is inevitable. In the next step, we optimized monoglucoside synthesis by applying Gtf180-ΔN mutants, aiming to increase the *K*_m_ value for catechol-G1 and/or decrease the *K*_m_ value for catechol.

### Mutational engineering of the Gtf180-ΔN enzyme

#### Selection of Gtf180-ΔN mutants

A library of 61 mutants with single amino acid residue changes (Table S1), targeting 10 amino acid residues of the Gtf180-ΔN acceptor binding sites +1 and +2, has been constructed previously (Meng et al. 2016; Meng et al. 2015). A quick and qualitative screening was performed to identify mutants displaying a relative increase in monoglycosylation and a decrease in α-glucan synthesis. For this purpose, 1 U/mL of every mutant was incubated for 1 h at the optimal reaction conditions (400 mM catechol, 1000 mM sucrose). The resulting reaction mixtures were subsequently spotted on TLC plates and mutually compared after staining (Figure S2).

Mutants of residues D1085, R1088, and N1089 were not affected in catechol glycosylation, since their product profiles were nearly identical to those of the WT Gtf180-ΔN. Mutants of W1065, a residue proven to be essential for both activity and acceptor binding by interacting with maltose through aromatic stacking (Leemhuis et al. 2012; Vujicic et al. 2010), displayed a very low total activity. Although the product profiles of these mutants were improved (more catechol-G1), their low total conversion and low specific activity rendered them less useful as glycosylation biocatalyst. Mutating D1028 yielded mutants with an enhanced oligosaccharide synthesis, as suggested by the more intense α-glucan oligosaccharide tail visible on TLC (Figure S2). Since this was the opposite of what was aimed for, these mutants were not selected for further analysis. Mutants of L940 all showed a shift in diglucoside linkage type, forming almost exclusively (α1 → 6) bonds. Indeed, the crucial role of L940 for linkage specificity in α-glucan synthesis was demonstrated previously (Meng et al. 2014). However, no relative increase in monoglycosylation yield was detected.

Mutants of residues L938, L981, and N1029 provided the most interesting results. Every L938 mutant tested showed an increased monoglucoside synthesis and a decreased formation of di- and triglucosides; the strongest effect was observed for mutant L938F (Figure S2). Similar effects were obtained with L981 mutants, especially when the leucine residue was replaced by alanine. In case of N1029 mutations, two different effects were observed. Firstly, when replacing asparagine by either glycine or threonine, almost exclusively (α1 → 3) diglucosides were synthesized, as was also seen for α-glucan synthesis (Meng et al. 2015). Secondly, when asparagine was replaced by methionine and to a lesser extent by tyrosine, the formation of di- and triglucosides was significantly reduced in favor of monoglucoside synthesis (Figure S2). From each mutant group, the best representative (L938F, L981A, and N1029M) was selected for further characterization and subjected to detailed analysis of products formed.

#### Characterization of Gtf180-ΔN mutants: increased catechol monoglycosylation

The L938F mutant displayed a higher total activity on sucrose as both acceptor and donor substrate than Gtf180-ΔN WT (132 %) at 1000 mM sucrose, whereas the L981A and N1029M mutants had reduced activity, retaining 23 and 32 % of the Gtf180-ΔN WT activity respectively (data not shown). To compare the mutants with WT Gtf180-ΔN, 4 U/mL of every Gtf180-ΔN mutant enzyme was incubated at optimal reaction conditions (400 mM catechol, 1000 mM sucrose), allowing analysis of the time-course synthesis of α-d-glucosides of catechol (Fig. 1b). The corresponding glycosylation yields and glucoside distributions are given in Table 3.

**Table 3:** Glycosylation yields and glucoside distribution of WT Gtf180-ΔN and mutants derived (400 mM catechol, 1000 mM sucrose, 4 U/mL Gtf180-ΔN)

	Catechol glucoside (mM)				Catechol glucoside distribution (%)		
Gtf180-ΔN	G1	G2_(α1 → 3)_	G2_(α1 → 6)_	G3+	G1	G2	G3
WT	241.1	33.8	68.0	57.3	60	25	15
L938F	311.3	51.8	10.0	26.9	78	15	7
N1029M	338.6	36.1	<10.0	19.6	85	10	5
L981A	373.5	<10.0	17.9	<10.0	93	6	1

*T* = 37 °C, pH = 4.7. All data given at maximal catechol-G1 yield (20 min incubation)

Similarly to Gtf180-ΔN WT, all mutants completely converted catechol into α-D-glucosides. However, the glucoside distribution was altered: the mutants displayed higher monoglycosylation yields. Up to 93 % catechol was converted into solely monoglucoside for the best performing mutant (L981A), compared to 60 % for Gtf180-ΔN. Interestingly, each of these mutants exhibited a shift in diglucoside linkage type compared to Gtf180-ΔN, favoring the formation of (α1 → 3) linkages (Table 3).

Determination of the kinetic parameters (Table 1) revealed that two opposite but related effects form the basis for the improved monoglycosylation yields. Except for the L938F mutant, the mutants had lower *k*_cat_ values for the acceptor reaction with catechol and sucrose (Table 1), mainly representing a reduction in total activity with sucrose alone as shown above. However, all mutants displayed much lower *K*_m_ values for catechol than the Gtf180-ΔN WT. In particular, the L981A mutant had a low *K*_m_ value of 11.0 mM for catechol, representing a 9-fold improvement compared to the Gtf180-ΔN WT. Despite the relatively low total activity of mutant L981A (23 % of Gtf180-ΔN), its catalytic efficiency (*k*_cat_/*K*_m_) for the acceptor reaction with catechol (plus sucrose) was 2.5-fold higher than of Gtf180-ΔN WT. The exact opposite was observed when comparing the kinetic parameters of the mutants with Gtf180-ΔN WT for catechol-G1 as acceptor substrate: all mutants displayed higher *K*_m_ values for catechol-G1 whereas their catalytic efficiencies were substantially lower.

To elucidate the underlying molecular mechanism, the transglycosylation and total activities of the Gtf180-ΔN (mutants), incubated with sucrose only, were determined. Subsequently, the α-glucan synthesis potential (α-GSP) was calculated, defined as the ratio of the transglycosylation activity over the total activity, revealing the potential of the enzyme to use the donor substrate sucrose for α-glucan synthesis (and not for hydrolysis). As shown in Table 1, the mutants showed a decrease in α-GSP compared to the Gtf180-ΔN WT.

In conclusion, mutant L981A represents a highly efficient biocatalyst for the glycosylation of catechol, yielding roughly 100 g/L catechol-G1 (373 mM) with a yield of 93 %.

#### Characterization of Gtf180-ΔN mutants: increased acceptor substrate conversion

**Fig. 2:**
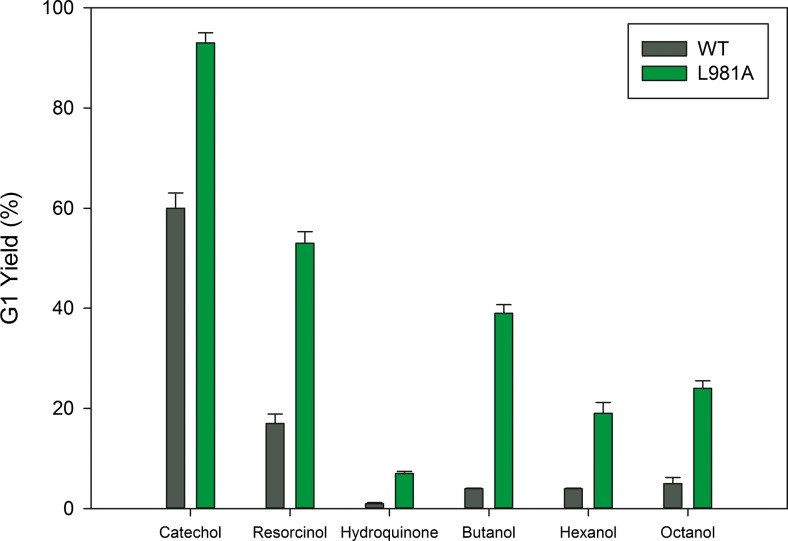
Monoglycosylation yields of WT Gtf180-ΔN and the L981A mutant derived (400 mM catechol/resorcinol/hydroquinone/butanol, 58 mM hexanol, 4 mM octanol, 1000 mM sucrose, 4 U/mL Gtf180-ΔN). All monoglycosylation yields represent maximum values (incubation time dependent on acceptor substrate). *T* = 37 °C, pH = 4.7

Due to an increased affinity for catechol which resulted from an impaired α-GSP, the monoglycosylation yield of Gtf180-ΔN mutants for the glycosylation of catechol was significantly improved. Subsequently, we determined whether the same effects could be observed when the L981A mutant was incubated with other acceptor substrates. Suppressing α-glucan synthesis by glucansucrase enzymes may provide a general strategy resulting in higher conversions of a wide range of non-carbohydrate acceptor substrates into α-d-glucosides, more specifically into monoglucosides. A diverse range of small non-carbohydrate molecules (resorcinol, hydroquinone, butanol, hexanol, octanol, pyridoxine, and resveratrol) were incubated with wild-type Gtf180-ΔN and the L981A mutant, plus sucrose. Indeed, compared to WT enzyme, the L981A mutant displayed increased monoglycosylation yields, from 17 to 53 % for resorcinol, 1 to 7 % for hydroquinone, 4 to 39 % for butanol, 4 to 19 % for hexanol, and 5 to 24 % for octanol (Fig. 2). To our knowledge, this is the first report of the enzymatic synthesis of hexyl- and octyl α-d-glucosides with a glucansucrase enzyme. Also, in the case of pyridoxine- and resveratrol glycosylation, an increase in monoglycosylation yield was observed by TLC analysis (not shown).

**Fig. 3:**
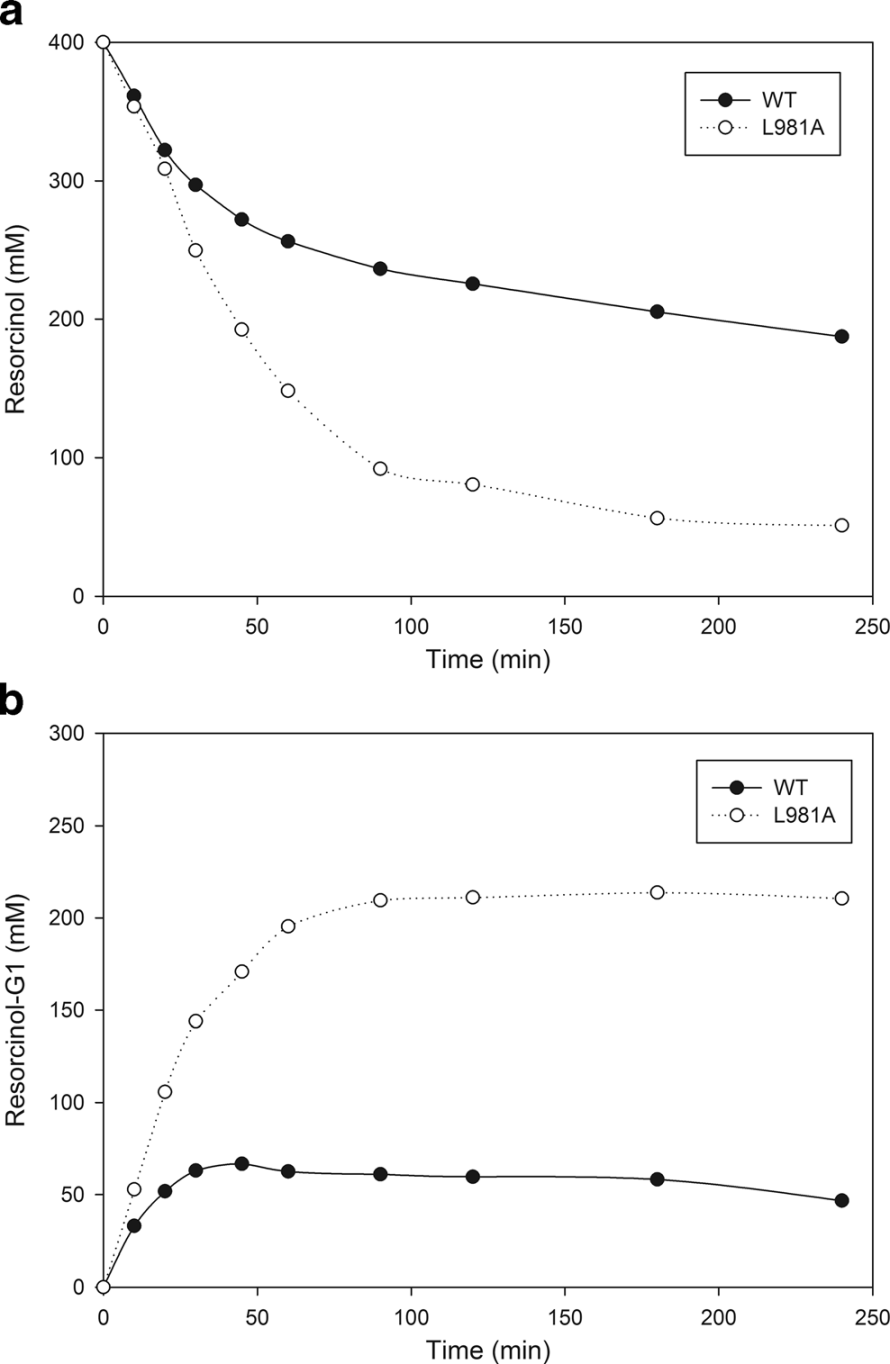
**a**, **b** Conversion of the resorcinol acceptor substrate and G1 production by WT Gtf180-ΔN and the L981A mutant derived (400 mM resorcinol, 1000 mM sucrose, 4 U/mL Gtf180-ΔN (mutant)). *T* = 37 °C, pH = 4.7

As illustrated for the glycosylation of resorcinol, two effects contributed to the enhancement of the monoglycosylation yields by the L981A mutant enzyme (Fig. 3). Firstly, the conversion of resorcinol acceptor substrate into α-d-glucosides was increased from 53 % by WT to 87 % by the mutant. Secondly, and similar to catechol glycosylation, the glycoside distribution was shifted towards mainly G1 production. With Gtf180-ΔN WT, 32 % of the glycosylated resorcinol consisted of monoglucoside after 4 h of incubation, whereas the L981A mutant had converted 61 % of the resorcinol into monoglucoside at *t* = 4 h. The production of monoglucoside reached its maximum long before the maximal resorcinol conversion (Fig. 3). The two effects of the mutagenesis are clearly illustrated by TLC analysis of the products obtained (Figure S3): during a 4 h incubation, L981A synthesized fewer oligo- and polysaccharides than Gtf180-ΔN. Instead, more resorcinol was converted into α-d-glucosides.

### Structural characterization of purified α-d-glucosides

**Fig. 4:**
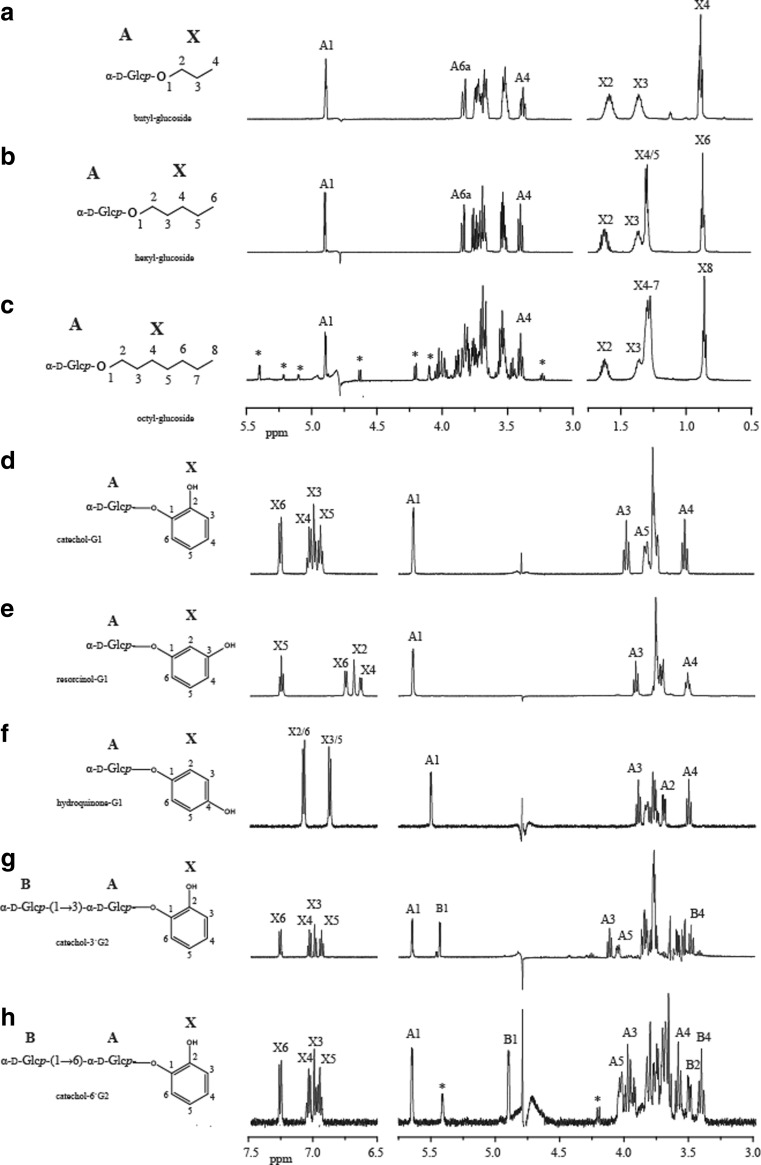
1D ^1^H NMR spectra of **a** butyl glucoside, **b** hexyl glucoside, **c** octyl glucoside, **d** catechol-G1, **e** resorcinol-G1, **f** hydroquinone-G1, **g** catechol-3′G2, and **h** catechol-6′G2

The biocatalytic synthesis of the α-d-glucosides of catechol, resorcinol, hydroquinone, butanol, hexanol, and octanol was confirmed by a combination of 1D NMR (^1^H NMR and ^13^C NMR) and 2D NMR spectroscopy. Figure 4 depicts the 1D ^1^H NMR spectra of the α-d-glucosides. The corresponding ^1^H and ^13^C chemical shifts are presented in the supplementary information (Tables S2 and S3). Figures S4–S9 of the supplementary information represent the 1D ^1^H NMR spectrum and 2D ^1^H–^1^H COSY, TOCSY (150 ms mixing time), ROESY (300 ms mixing time), and ^13^C–^1^H HSQC spectra of butyl glucoside, hexyl glucoside, octyl glucoside, resorcinol-G1, hydroquinone-G1, and catechol-3′G2, respectively. The 1D ^1^H NMR spectra of catechol-G1 and catechol-6′G2 matched with those found previously by te Poele et al. (2016) and are presented there. For a detailed analysis of the NMR spectra, see supplementary information.

## Discussion

In view of their broad acceptor substrate specificity, glucansucrases are considered promising glycosylation biocatalysts. However, the typical synthesis of a mixture of α-d-glucosides, oligosaccharides, and α-d-glucans remains a bottleneck in their industrial application. α-Glucan synthesis is the main glucansucrase reaction but an undesired side reaction when aiming to glycosylate non-carbohydrate acceptor substrates, lowering glycosylation yields and complicating downstream processing. For example, when applying salicin and salicyl alcohol as acceptor substrates, B-1355C2 and B-1299CB-BF563 dextransucrases from *Leuconostoc mensenteroides* synthesized at least 12 and 9 different kinds of glycosides, respectively (Seo et al. 2005).

So far, few enzyme engineering studies with glucansucrases have focused on glycosylation of non-carbohydrate acceptor substrates. In 2014, Malbert et al. reported a significant improvement of luteolin monoglycosylation by the I228A *Np*AS mutant compared to the wild-type enzyme. Docking studies attributed this enhancement to the introduction of a less hindering residue, assisting in a better positioning of luteolin in the catalytic pocket. In 2016, Liang et al. expanded the acceptor substrate promiscuity of GtfD from *Streptococcus mutans* by simultaneous site-saturation mutagenesis of residues Y418 and N469. The best mutant (Y418R and N469C) exhibited a significant improvement in transglycosylation activities towards several flavonoids, the major products being monoglucosylated. Docking studies were based on the crystal structure of Gtf180-ΔN and revealed three additional hydrogen bonds with the flavonoid acceptor substrate compared to the wild type, resulting in the increased catalytic efficiency of the mutant enzyme. Recently, two substantial improvements were made in sucrose phosphorylase mediated glycosylation of phenolic compounds. The enhanced performance was realized by the construction of mutants with a better accessibility of the active site (Dirks-Hofmeister et al. 2015; Kraus et al. 2016).

In the present study, the aim was to improve glycosylation yields by suppressing the competing α-glucan synthesis reaction, rather than engineering the active site to make it more suitable for non-carbohydrate acceptor substrates. As presented in the “Results” section, this resulted in a strong optimization of monoglycosylated product synthesis by the glucansucrase Gtf180-ΔN. The model acceptor substrate catechol was almost completely glycosylated into monoglycosylated product by the L981A mutant (93 % compared to 60 % for the wild-type enzyme), substantially higher than previously reported for catechol glycosylation by GtfD from *S. mutans* (65 %) (Meulenbeld and Hartmans 2000). In comparison, the I228A *Np*AS mutant only displayed a luteolin monoglycosylation yield of 53 % (Malbert et al. 2014), whereas the GtfD mutant showed a catechin monoglycosylation yield of 90 % (Liang et al. 2016).

**Fig. 5:**
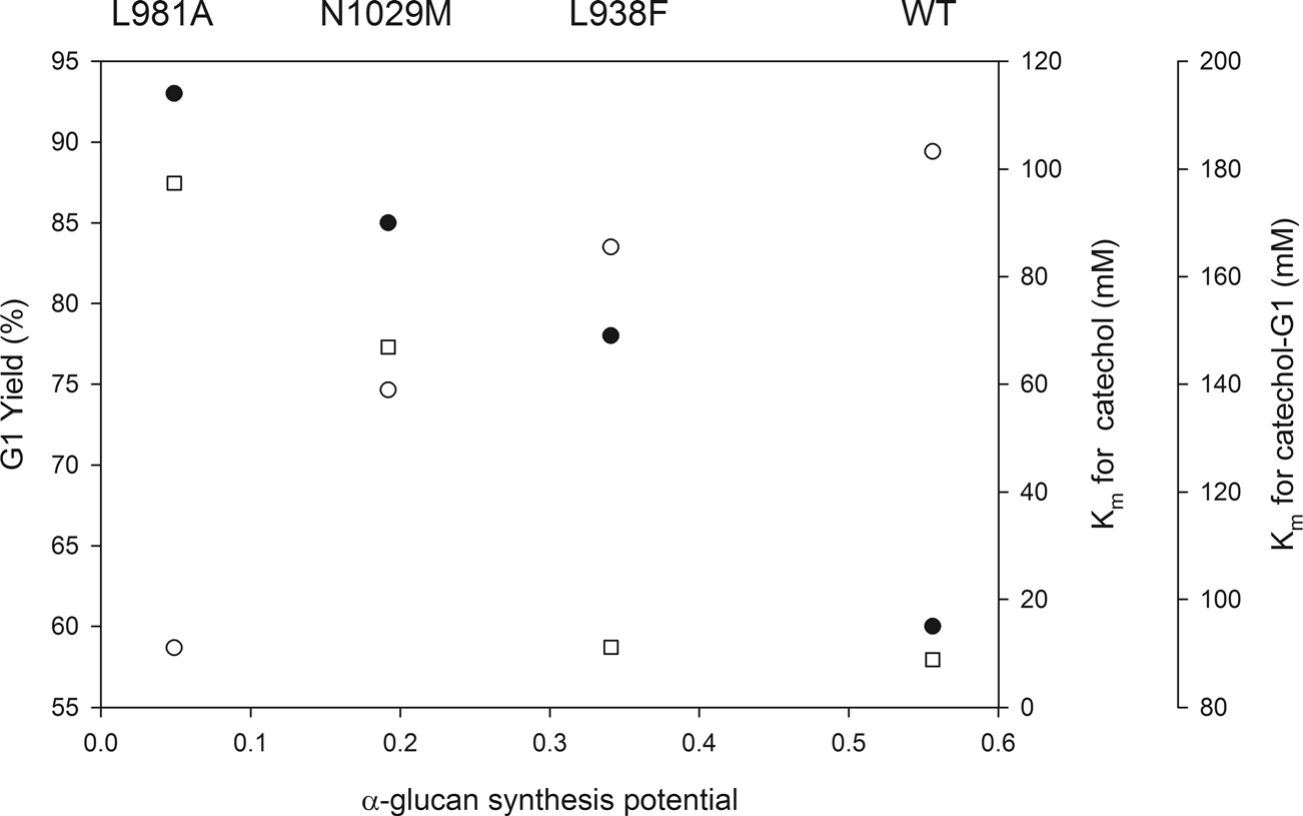
Correlation between α-GSP for sucrose as acceptor substrate, *K*_m_ for catechol and catechol-G1 as acceptor substrate, and G1 yield of WT Gtf180-ΔN and mutants derived. Data are listed in Tables 1 and 3. *Filled circles* G1 yield (%), *unfilled circles K*_m_ for catechol (mM), *unfilled squares K*_m_ for catechol-G1 (mM)

Kinetic analysis indicated that the Gtf180-ΔN mutants (partly) lost their ability to synthesize α-glucan polysaccharides, as was previously shown by Meng et al. (2015). However, this positively influenced the glycosylation of catechol. Indeed, a positive correlation could be established between α-GSP and the *K*_m_ values for catechol, whereas a negative correlation was found between α-GSP and the monoglycosylation yields with catechol (Fig. 5). This shows that these mutations (partly) suppressed the competing α-glucan synthesis, yielding mutants with an improved affinity for catechol as acceptor substrate. Moreover, the increased *K*_m_ values of these mutants for catechol-G1 also revealed a reduced α-GSP. Indeed, in the active site of glucansucrase enzymes, α-d-glucosides will basically behave like saccharides. Therefore, the affinities of these mutants for catechol-G1 and for saccharides are positively correlated. The combination of an improved affinity for catechol with a decreased affinity for catechol-G1 thus resulted in the higher monoglycosylation yields of these Gtf180-ΔN mutants.

Moreover, suppressing α-glucan synthesis by mutagenesis of Gtf180-ΔN (L981A) clearly resulted in improved monoglycosylation yields for all the phenolic and alcoholic compounds tested here. Mutagenesis of the +1 and +2 acceptor substrate binding sites thus provides a general strategy to improve the monoglycosylation yields of non-carbohydrate acceptor substrates of glucansucrase enzymes.

**Fig. 6:**
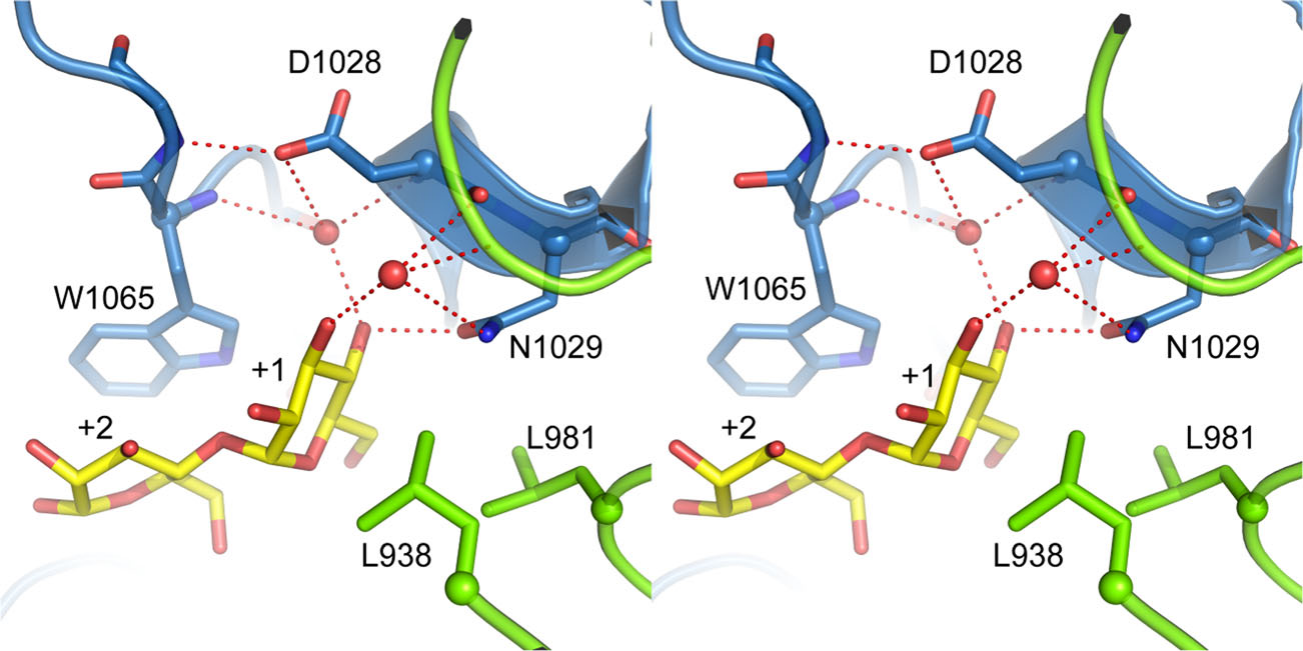
Stereo view of Gtf180-ΔN with the acceptor maltose (*yellow carbon atoms*) bound in subsites +1 and +2 (PDB: 3KLL). Residue N1029 from domain A (*blue*) provides direct and indirect (water-mediated) hydrogen bonds to the non-reducing end glucosyl unit bound at subsite +1. Residues L938 and L981 from domain B (*green*) are also near subsite +1. This figure has been adapted from Meng et al. (2015)

The architecture of the +1 acceptor substrate binding site is the main determinant of whether an acceptor substrate will bind to the active site or not and consequently react with the covalently attached glucosyl moiety (Leemhuis et al. 2012). The crystal structure of Gtf180-ΔN in complex with maltose, representing a typical saccharide acceptor substrate (Vujicic-Zagar et al. 2010), was studied with the aim of understanding how mutagenesis of the discussed residues impairs α-glucan synthesis (Fig. 6). Firstly, N1029 interacts with the non-reducing end glucosyl moiety of maltose by means of direct and indirect hydrogen bonds with the C4 and C3 hydroxyl groups. Mutating the asparagine to a methionine removes this interaction, lowering the affinity of the enzyme for maltose. Hence, α-glucan synthesis is suppressed which improves the glycosylation of non-carbohydrate acceptor substrates. In contrast, L981 and L938 do not provide maltose with hydrogen bond interactions. Due to their hydrophobic nature, they contribute by shaping the active site near subsite +1. Introducing an alanine at position 981 presumably reduces the hydrophobic interaction with the C6 of the non-reducing end glucosyl moiety of maltose. Apparently, this severely impairs α-glucan synthesis yielding a Gtf180-ΔN variant with enhanced glycosylation of non-carbohydrate acceptor substrates. On the other hand, mutating L938 to a bulky residue like phenylalanine partially blocks the +1 subsite thereby preventing maltose to efficiently interact with the other residues. This has a smaller effect on α-glucan synthesis than L981A and N1029M, resulting in a limited improvement of the glycosylation of non-carbohydrate acceptor substrates.

In conclusion, by applying the optimal reaction conditions and using the best Gtf180-ΔN mutant, a wide range of non-carbohydrate acceptor substrates could more efficiently be converted into mainly monoglycosylated products. Consequently, the glycosylation potential of the Gtf180-ΔN enzyme was strongly improved. Furthermore, the screening strategy applied in this paper yielded mutants that can be used as templates to further engineer the Gtf180-ΔN active site for improved glycosylation of specific acceptor substrates.

## Electronic supplementary material

ESM 1 (PDF 1006 kb)
