# Supplementary material for: Glucansucrase Gtf180-ΔN of Lactobacillus reuteri 180: enzyme and reaction engineering for improved glycosylation of non-carbohydrate molecules
Source: Appl Microbiol Biotechnol. 2016 Apr 6;100:7529–39. doi: 10.1007/s00253-016-7476-x (PMC4980424; doi:10.1007/s00253-016-7476-x)
Supplement: Supplementary file 1 — (PDF 1006 kb) [file 253_2016_7476_MOESM1_ESM.pdf]

Applied Microbiology and Biotechnology

Glucansucrase Gtf180-ΔN of *Lactobacillus reuteri* 180: Enzyme and reaction engineering for improved glycosylation of non-carbohydrate molecules

Tim Devlamynck[1,2], Evelien M. te Poele[1], Xiangfeng Meng[1],
Sander S. van Leeuwen[1] and Lubbert Dijkhuizen[1]

[1]Microbial Physiology, Groningen Biomolecular Sciences and Biotechnology Institute (GBB) University of Groningen, Nijenborgh 7, 9747 AG Groningen, The Netherlands

[2]Centre for Industrial Biotechnology and Biocatalysis, Department of Biochemical and Microbial Technology, Faculty of Bioscience Engineering, Ghent University, Coupure Links 653, 9000 Ghent, Belgium

Corresponding author: l.dijkhuizen@rug.nl

Supplementary information

1. Detailed analysis of the NMR spectra

1.1. Alkyl glucosides

The 1D ^{1}H NMR spectrum of the isolated product from the incubation with butanol (Figure S4) showed one anomeric signal at δ 4.901 ppm (**A** H-1; $^{3}J_{1,2}$ 3.91 Hz), indicative of an α-anomeric residue. The signal at δ 0.908 (**X** H-4, *t*) with an intensity corresponding with 3 protons, fits with the butanol CH_3 signal. Starting from **A** H-1 for the glucose moiety and **X** H-4 for the butanol moiety, all ^{1}H chemical shifts with their corresponding ^{13}C chemical shifts could be determined from 2D ^{1}H-^{1}H and 2D ^{13}C-^{1}H NMR spectra (Table S2, Figure S4). The pattern of ^{1}H and ^{13}C chemical shifts fits with a non-reducing terminal α-D-Glc*p*-residue (Van Leeuwen et al. 2008). Due to the influence of the Glc-moiety linked to the butanol the H-1 protons are shifted to **X** H-1a (δ 3.73) and **X** H-1b (δ 3.53). The 2D ^{1}H-^{1}H ROESY spectrum (Figure S4) showed correlations between **A** H-1 and **X** H-1a and between **A** H-1 and **X** H-2. These data confirm the successful coupling of a Glc-residue to butanol, via an α-linkage.

The 1D ^{1}H NMR spectrum of the isolated product of the reaction with hexanol (Figure S5) showed a pattern similar to that of α-D-Glc*p*-butanol, with an anomeric signal at δ 4.903 (**A** H-1; $^{3}J_{1,2}$ 3.87 Hz), fitting with an α-anomeric residue. The signal at δ 0.868 (**X** H-6, *t*), with an intensity corresponding with 3 protons fits with the hexanol CH_3 signal. Using 2D ^{1}H-^{1}H and 2D ^{13}C-^{1}H NMR spectroscopy all ^{1}H and ^{13}C chemical shifts were determined (Table S2, Figure S5). The pattern for residue **A** fits again with a non-reducing terminal α-D-Glc*p*-residue. The 2D ^{1}H-^{1}H ROESY spectrum revealed correlations between **A** H-1 and **X** H-1a and between **A** H-1 and **X** H-2, confirming the coupling of an α-D-Glc*p*-residue to hexanol.

The 1D ^{1}H NMR spectrum of the isolated product of the reaction with octanol (Figure S6) showed a more complex pattern of peaks. Here the signal for **A** H-1 was observed at δ 4.898 ($^{3}J_{1,2}$ 3.76 Hz), similar to the butanol and hexanol products. The octanol CH_3 signal (**X** H-8) is found at δ 0.855 (*t*). Using 2D NMR spectroscopy all ^{1}H and ^{13}C chemical shifts for **A** and **X** were found (Table S2, Figure S6). The patterns for residue **A** and **X** are again comparable to those of the other alkyl glucosides. The coupling of the α-D-Glc*p* residue is confirmed by 2D ^{1}H-^{1}H ROESY cross-peaks between **A** H-1 and **X** H-1a and **A** H-1 and **X** H-2.

1.2. Benzenediol glucosides

The 1D ^{1}H NMR spectra of the reaction products catechol-G1 and catechol-6`G2 from the reaction with catechol match with those found previously for α-D-Glc*p*-catechol and α-D-Glc*p*-(1→6)-α-D-Glc*p*-catechol, respectively (te Poele et al. 2016). All ^{1}H and ^{13}C chemical shifts are presented in Table S3.

The 1D ^{1}H NMR spectrum of the structure isolated from the reaction with resorcinol as acceptor (Figure S7) showed one anomeric signal at δ 5.634 (**A1**; $^{3}J_{1,2}$ 3.78 Hz) indicating an α-linked residue. Using 2D ^{1}H-^{1}H and ^{13}C-^{1}H NMR spectroscopy all ^{1}H and ^{13}C chemical shifts were assigned (Table S3, Figure S7). Compared to free Glc (van Leeuwen et al. 2008a) the glucose H-2 signal is shifted significantly downfield (δ 3.722), probably as a result of interactions with the resorcinol aromatic ring, as observed previously for catechol glucoside (te Poele et al. 2016). The pattern of ^{1}H and ^{13}C chemical shifts of residue **A** fit with a non-reducing terminal α-D-Glc*p*-residue. In the 2D ^{1}H-^{1}H ROESY spectrum (Figure S7) interactions are observed between **A** H-1 and **X** H-2 and between **A** H-1 and **X** H-6, confirming the successful coupling of α-D-Glc*p* to resorcinol.

The 1D ^{1}H NMR spectrum of the product isolated from the reaction with hydroquinone (Figure S8) showed one α-anomeric signal at δ 5.490 (**A** H-1; $^{3}J_{1,2}$ 3.62 Hz) and hydroquinone signals at δ 7.078 (**X** H-2 and H-6) and at δ 6.871 (**X** H-3 and H-5). Using 2D ^{1}H-^{1}H and ^{13}C-^{1}H NMR spectroscopy all ^{1}H and their corresponding ^{13}C chemical shifts were determined (Table S3, Figure S8). The pattern of ^{13}C chemical shifts of residue **A** fits with a terminal α-D-Glc*p*-residue. The ^{1}H chemical shifts of residue **A** fit best with a residue linked to an aromatic moiety, note **A** H-2 at δ 3.75, which is significantly downfield, as was observed for the catechol and resorcinol glucosides as well. The successful coupling of α-D-Glc*p* to hydroquinone is further supported by the 2D ^{1}H-^{1}H ROESY correlations (Figure S8) between **A** H-1 and **X** H-2 and H-6.

The 1D ^{1}H NMR spectrum of the third structure isolated from the incubation with catechol (Figure S9) showed two α-anomeric signals at δ 5.635 (**A** H-1; $^{3}J_{1,2}$ 3.76 Hz) and δ 5.421 (**B** H-1; $^{3}J_{1,2}$ 3.83 Hz), fitting with two α-D-Glc*p*-residues. All ^{1}H and their corresponding ^{13}C chemical shifts were determined from 2D NMR spectra (Table S3, Figure S9). The pattern of

chemical shifts for residue **A** showed significant downfield shifts of **A** H-2 (δ 3.86; Δδ + 0.11), **A** H-3 (δ 4.116; Δδ + 0.13) and **A** H-4 (δ 3.79; Δδ + 0.25), compared with residue **A** in α-D-Glc*p*-catechol (Table S3). This fits best with a 3-substitution of residue **A** (Van Leeuwen et al. 2008). Residue **B** has a pattern of ^{1}H chemical shifts fitting with a terminal residue involved in an (α1→3)-linkage. The position of **B** H-5 at δ 4.050, significantly downfield compared to terminal residues involved in a (α1→4) or (α1→6)-linkage (δ 3.73-3.76) (Van Leeuwen et al. 2008) is typical for such a residue. The 3-substitution of residue **A** is also reflected in the ^{13}C chemical shift of C-3, significantly downfield at δ 80.6 (Bock and Thøgersen, 1982). Furthermore, the 2D ^{1}H-^{1}H ROESY spectrum (Figure S9) showed correlations between **B** H-1 and **A** H-3 and between **A** H-1 and **X** H-6, confirming the structure as α-D-Glc*p*-(1→3)-α-D-Glc*p*-catechol.

References

Bock K, Thøgersen H (1982) Nuclear magnetic resonance spectroscopy of mono- and oligosaccharides. Annu Rep NMR Spectrosc 13:2-57.

Meng X, Dobruchowska JM, Pijning T, Gerwig GJ, Dijkhuizen L (2015a) Synthesis of new hyper-branched α-glucans from sucrose by *Lactobacillus reuteri* 180 glucansucrase mutants. J Agr Food Chem, in press. doi: 10.1021/acs.jafc.5b05161

Meng X, Pijning T, Dobruchowska JM, Gerwig GJ, Dijkhuizen L (2015b) Characterization of the functional roles of amino acid residues in acceptor binding subsite +1 in the active site of the glucansucrase GTF180 enzyme of *Lactobacillus reuteri* 180. J Biol Chem 290:30131-30141.

Van Leeuwen SS, Leeflang BR, Gerwig GJ, Kamerling JP (2008) Development of a ^{1}H NMR structural-reporter-group concept for the primary structural characterisation of α-D-glucans. Carb Res 343:1114-1119.

Tables

Table S1. List of mutants[1] of Gtf180-ΔN screened for their glycosylation potential.

**1**	L938A	**14**	A978F	**27**	D1028G	**40**	D1085Q	**53**	N1089D
**2**	L938S	**15**	A978S	**28**	D1028N	**41**	R1088H	**54**	N1089P
**3**	L938F	**16**	A978G	**29**	N1029Y	**42**	R1088K	**55**	W1065F
**4**	L938K	**17**	A978L	**30**	N1029G	**43**	R1088E	**56**	W1065K
**5**	L938M	**18**	A978P	**31**	N1029T	**44**	R1088W	**57**	W1065L
**6**	L940A	**19**	A978Y	**32**	N1029M	**45**	R1088T	**58**	W1065Q
**7**	L940S	**20**	L981A	**33**	N1029R	**46**	R1088N	**59**	W1065E
**8**	L940E	**21**	L981N	**34**	D1085Y	**47**	R1088G	**60**	W1065M
**9**	L940F	**22**	L981E	**35**	D1085V	**48**	N1089Y	**61**	W1065G
**10**	L940W	**23**	D1028Y	**36**	D1085A	**49**	N1089G		
**11**	L940G	**24**	D1028W	**37**	D1085E	**50**	N1089S		
**12**	L940M	**25**	D1028L	**38**	D1085H	**51**	N1089L		
**13**	L940C	**26**	D1028K	**39**	D1085L	**52**	N1089R		

[1]For construction and partial characterization of these mutants, see Meng et al. (2015a and b).

Table S2. ^{1}H and ^{13}C chemical shifts of alkyl glucosides, relative to internal acetone ($\delta^{1}H$ 2.225, $\delta^{13}C$ 31.08).

	**But-G1**		**Hex-G1**		**Oct-G1**	
	$\delta\ ^{1}H$	$\delta\ ^{13}C$	$\delta\ ^{1}H$	$\delta\ ^{13}C$	$\delta\ ^{1}H$	$\delta\ ^{13}C$
A 1	4.901	98.8	4.903	98.8	4.898	98.7
A 2	3.52	72.0	3.540	72.0	3.54	72.0
A 3	3.69	73.9	3.694	73.8	3.70	73.8
A 4	3.398	70.2	3.402	70.3	3.401	70.1
A 5	3.68	72.4	3.68	72.4	3.68	72.5
A 6a	3.845	61.2	3.844	61.1	3.840	61.1
A 6b	3.75		3.758		3.76	
X 1a	3.73	68.8	3.73	69.1	3.72	69.0
X 1b	3.53	68.8	3.53	69.1	3.53	69.0
X 2	1.613	31.4	1.629	29.3	1.621	29.2
X 3	1.378	19.5	1.366	35.9	1.354	26.1
X 4	0.908	13.8	1.30	31.7	1.27	31.9
X 5	-	-	1.30	22.7	1.28	29.0
X 6	-	-	0.868	14.1	1.30	29.0
X 7	-	-	-	-	1.30	22.8
X 8	-	-	-	-	0.855	13.9

Table S3. ^{1}H and ^{13}C chemical shifts of glucosides of benzenediols, relative to internal acetone (δ^{1}H 2.225, δ^{13}C 31.08).

	Res-G1		HQ-G1		Cat-G1		Cat-3`G2		Cat-6`G2	
	δ ^{1}H	δ ^{13}C	δ ^{1}H	δ ^{13}C	δ ^{1}H	δ ^{13}C	δ ^{1}H	δ ^{13}C	δ ^{1}H	δ ^{13}C
A 1	5.634	97.8	5.490	99.2	5.626	99.0	5.635	99.1	5.648	99.1
A 2	3.722	72.1	3.701	72.0	3.75	72.2	3.86	70.8	3.78	72.3
A 3	3.916	73.8	3.898	73.9	3.986	73.9	4.116	80.6	3.984	73.9
A 4	3.518	70.4	3.510	70.3	3.536	70.2	3.79	70.6	3.596	70.1
A 5	3.76	73.4	3.839	73.2	3.84	73.5	3.85	73.1	4.043	71.9
A 6a	3.78	61.1	3.798	61.1	3.81	61.2	3.83	61.1	3.70	66.4
A 6b	3.74		3.757		3.78		3.76		3.944	
B 1	-	-	-	-	-	-	5.421	100.3	4.901	98.5
B 2	-	-	-	-	-	-	3.591	72.7	3.508	72.4
B 3	-	-	-	-	-	-	3.79	73.4	3.668	73.9
B 4	-	-	-	-	-	-	3.485	70.2	3.419	70.4
B 5	-	-	-	-	-	-	4.050	72.7	3.72	72.9
B 6a	-	-	-	-	-	-	3.86	61.1	3.827	61.5
B 6b	-		-		-		3.80		3.76	
X 2	6.689	105.2	7.078	120.0	-	-	-	-	-	-
X 3	-	-	6.871	117.1	6.995	117.8	6.992	117.8	7.015	117.8
X 4	6.737	111.1	-	-	7.041	124.8	7.040	124.8	7.056	124.9
X 5	7.258	131.7	6.871	117.1	6.951	121.9	6.944	121.6	6.974	121.7

X 6	6.754	110.0	7.078	120.0	7.266	118.2	7.268	118.1	7.274	118.3

Figures

Fig. S1 Lineweaver-Burk plots for the glycosylation of catechol and catechol-G1 with Gtf180-ΔN. R^2 is 0.97 and 0.99, respectively. The corresponding kinetic data are listed in Table I.

Fig. S2 TLC analysis of glycosylated products synthesized by Gtf180-ΔN and mutants derived after 1 h of incubation (400 mM catechol; 1000 mM sucrose; 1 U/mL Gtf180-ΔN (mutants)). Numbers refer to mutants listed in Table I. G1 and G2 refer to the mono- and diglycosylated catechol products. Upper G2 spot: diglycosylated product with (α1→3) bond. Lower G2 spot: diglycosylated product with (α1→6) bond.

Fig. S3 TLC analysis of products of resorcinol glycosylation by WT Gtf180-ΔN and the L981A mutant derived (400 mM resorcinol; 1000 mM sucrose; 4 U/mL Gtf180-ΔN (mutant)). G1 and G2 refer to the mono- and diglycosylated resorcinol products.

Fig. S4 1D ^{1}H NMR spectrum, and 2D ^{1}H-^{1}H COSY, TOCSY (150 ms mixing time), ROESY (300 ms mixing time) and ^{13}C-^{1}H HSQC spectra of butyl glucoside.

Fig. S5 1D ^{1}H NMR spectrum, and 2D ^{1}H-^{1}H COSY, TOCSY (150 ms mixing time), ROESY (300 ms mixing time) and ^{13}C-^{1}H HSQC spectra of hexyl glucoside.

Fig. S6 1D ^{1}H NMR spectrum, and 2D ^{1}H-^{1}H COSY, TOCSY (150 ms mixing time), ROESY (300 ms mixing time) and ^{13}C-^{1}H HSQC spectra of octyl glucoside.

Fig. S7 1D ^{1}H NMR spectrum, and 2D ^{1}H-^{1}H COSY, TOCSY (150 ms mixing time), ROESY (300 ms mixing time) and ^{13}C-^{1}H HSQC spectra of resorcinol-G1.

Fig. S8 1D ^{1}H NMR spectrum, and 2D ^{1}H-^{1}H COSY, TOCSY (150 ms mixing time), ROESY (300 ms mixing time) and ^{13}C-^{1}H HSQC spectra of hydroquinone-G1.

Fig. S9 1D ^{1}H NMR spectrum, and 2D ^{1}H-^{1}H COSY, TOCSY (150 ms mixing time), ROESY (300 ms mixing time) and ^{13}C-^{1}H HSQC spectra of catechol-3`G2.

Figure S1

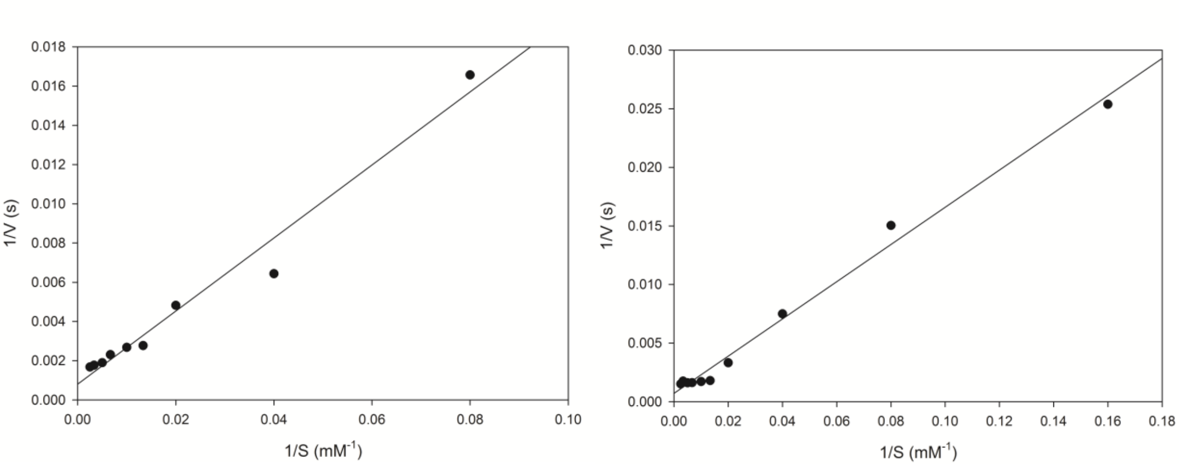

Figure S2

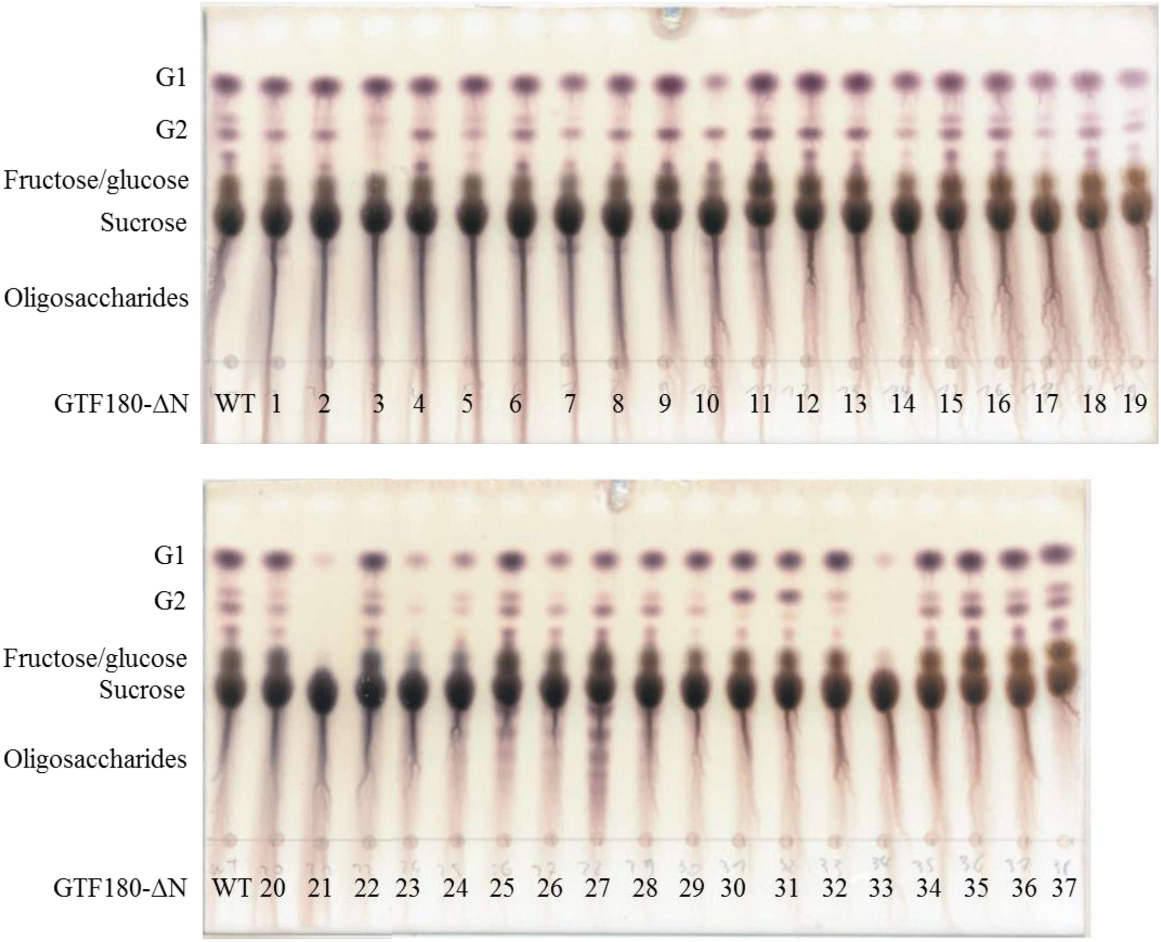

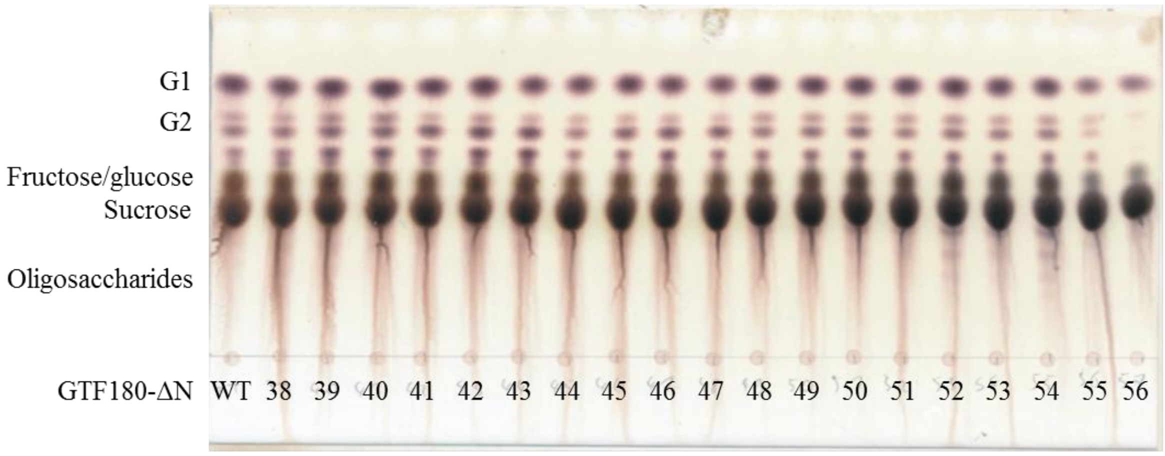
G1
G2
Fructose/glucose
Sucrose
Oligosaccharides
GTF180-ΔN WT 38 39 40 41 42 43 44 45 46 47 48 49 50 51 52 53 54 55 56

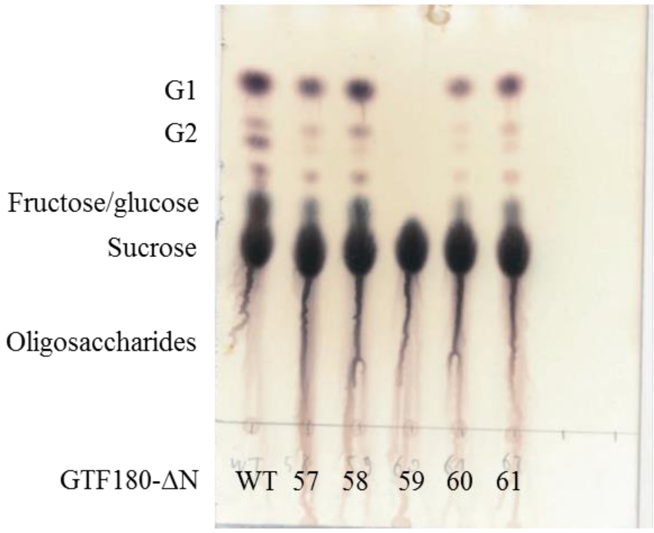
G1
G2
Fructose/glucose
Sucrose
Oligosaccharides
GTF180-ΔN WT 57 58 59 60 61

Figure S3

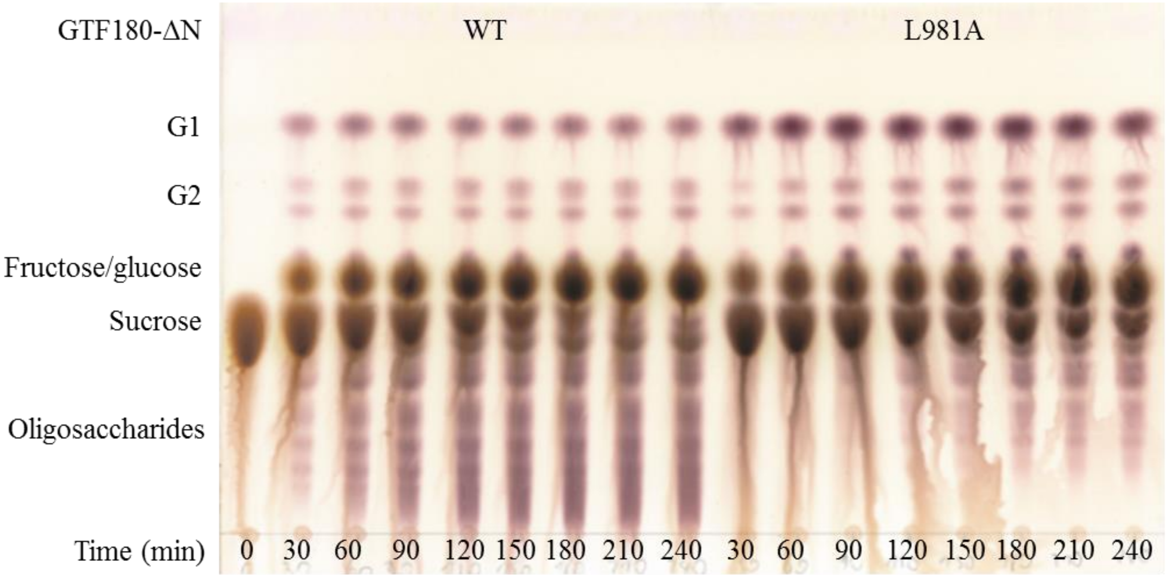
GTF180-ΔN
WT
L981A
G1
G2
Fructose/glucose
Sucrose
Oligosaccharides
Time (min) 0 30 60 90 120 150 180 210 240 30 60 90 120 150 180 210 240

Figure S4

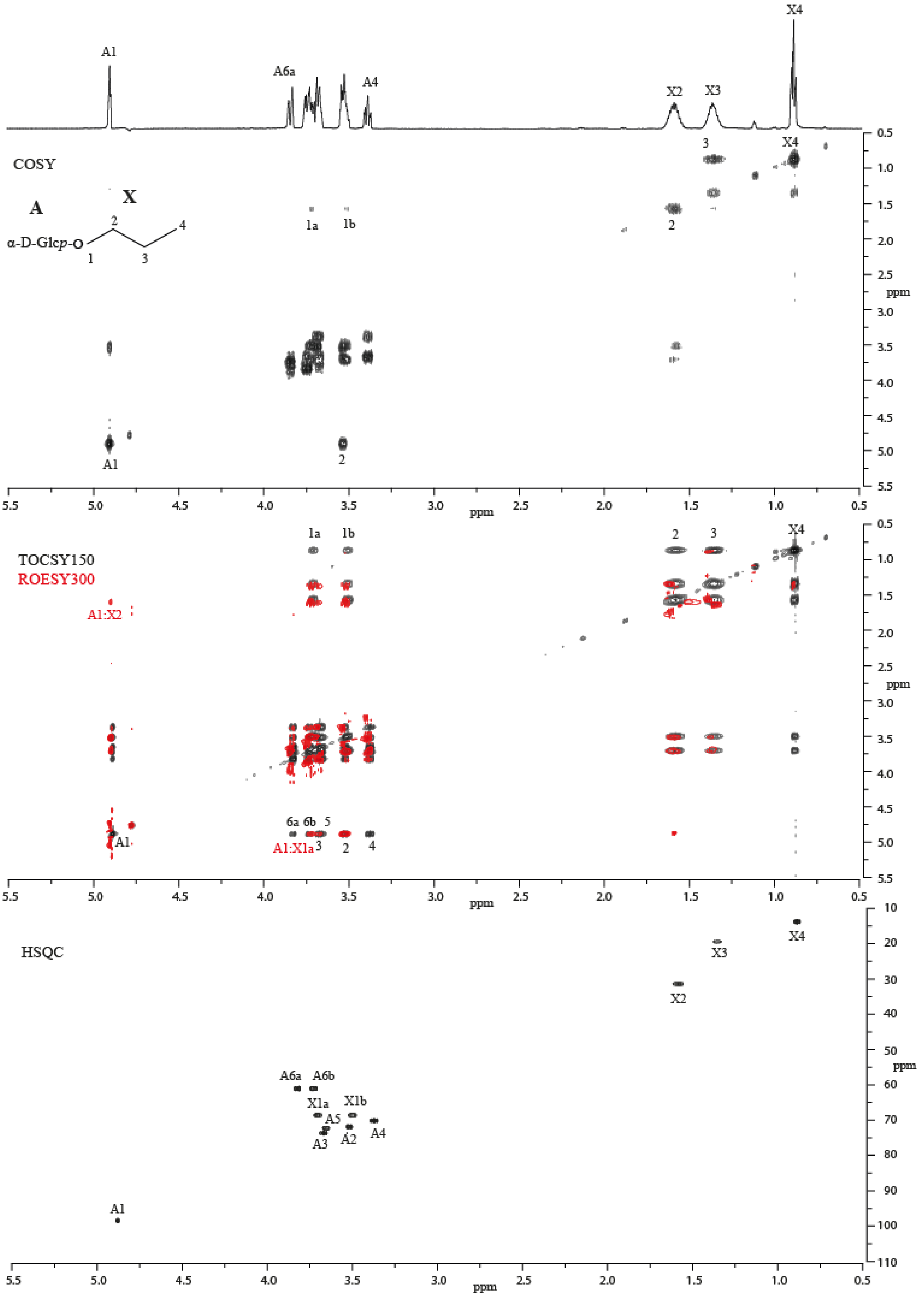
A1
A6a
A4
X2
X3
X4
COSY
A
X
α-D-Glcp-O
1
2
3
4
1a
1b
A1
TOCSY150
ROESY300
A1:X2
6a 6b 5
A1:X1a
HSQC
A6a A6b
X1a
X1b
A5
A3
A2
ppm

Figure S5

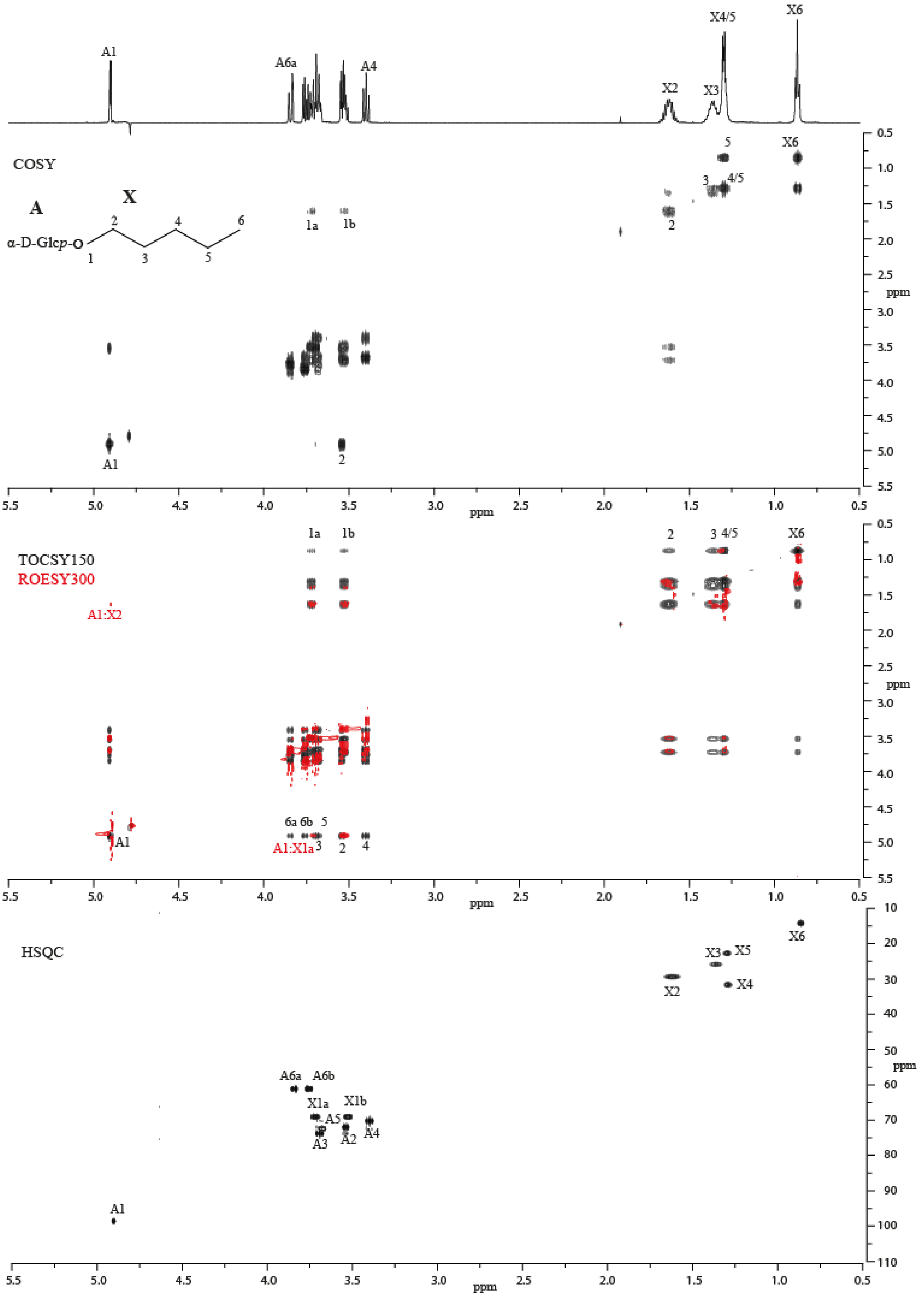

A1
A6a
A4
X2
X3
X4/5
X6
COSY
A
X
α-D-Glcp-O
1a
1b
TOCSY150
ROESY300
A1:X2
6a 6b 5
A1:X1a
HSQC
A6a A6b
X1a
X1b
A5
A3
A2
ppm

Figure S6

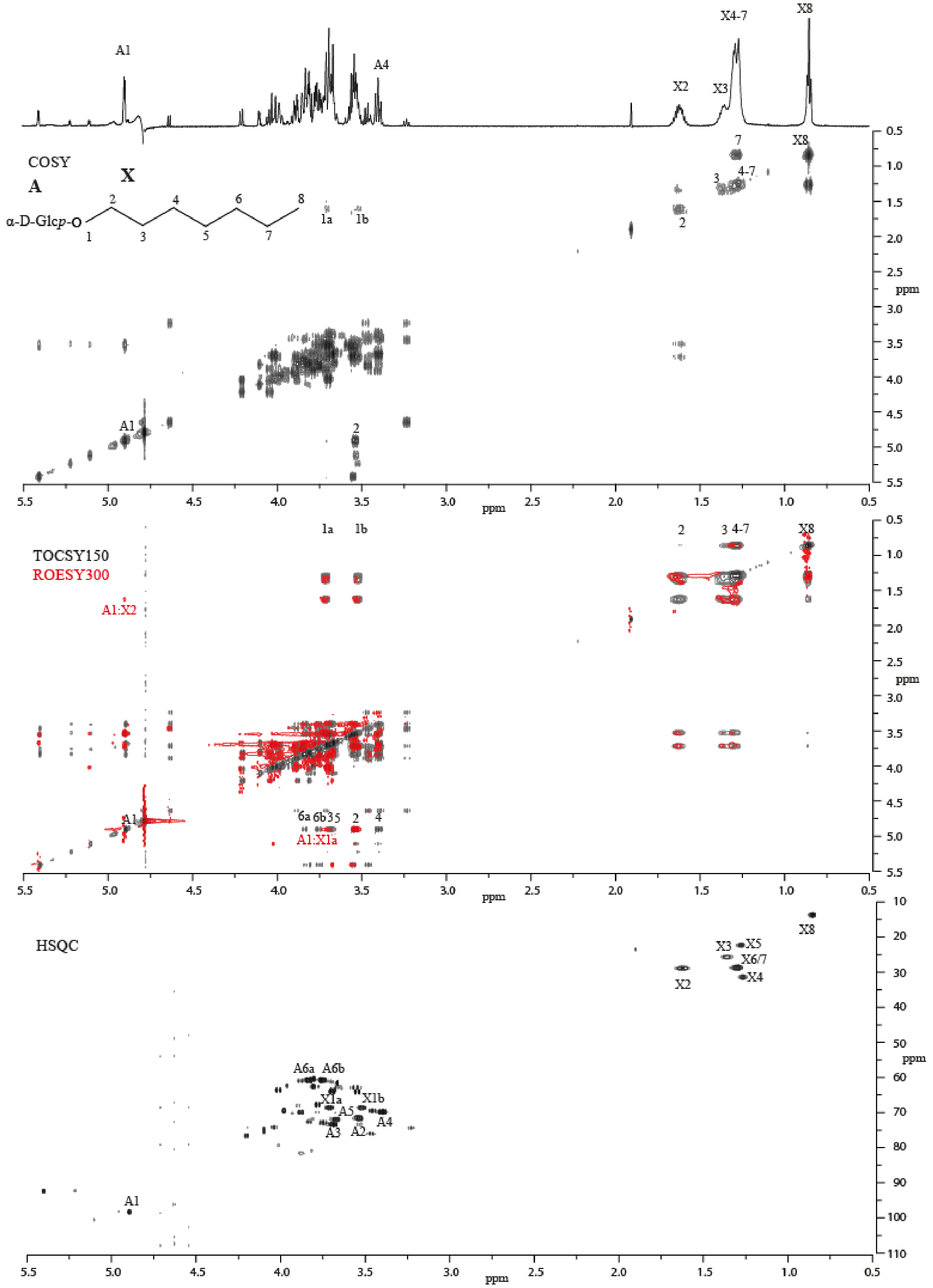
A1
A4
X2
X3
X4-7
X8
COSY
A
X
α-D-Glcp-O
1a
1b
TOCSY150
ROESY300
A1:X2
A1:X1a
6a 6b3 5
HSQC
A6a A6b
X1a
X1b
A5
A4
A3
A2
X5
X6/7
ppm

Figure S7

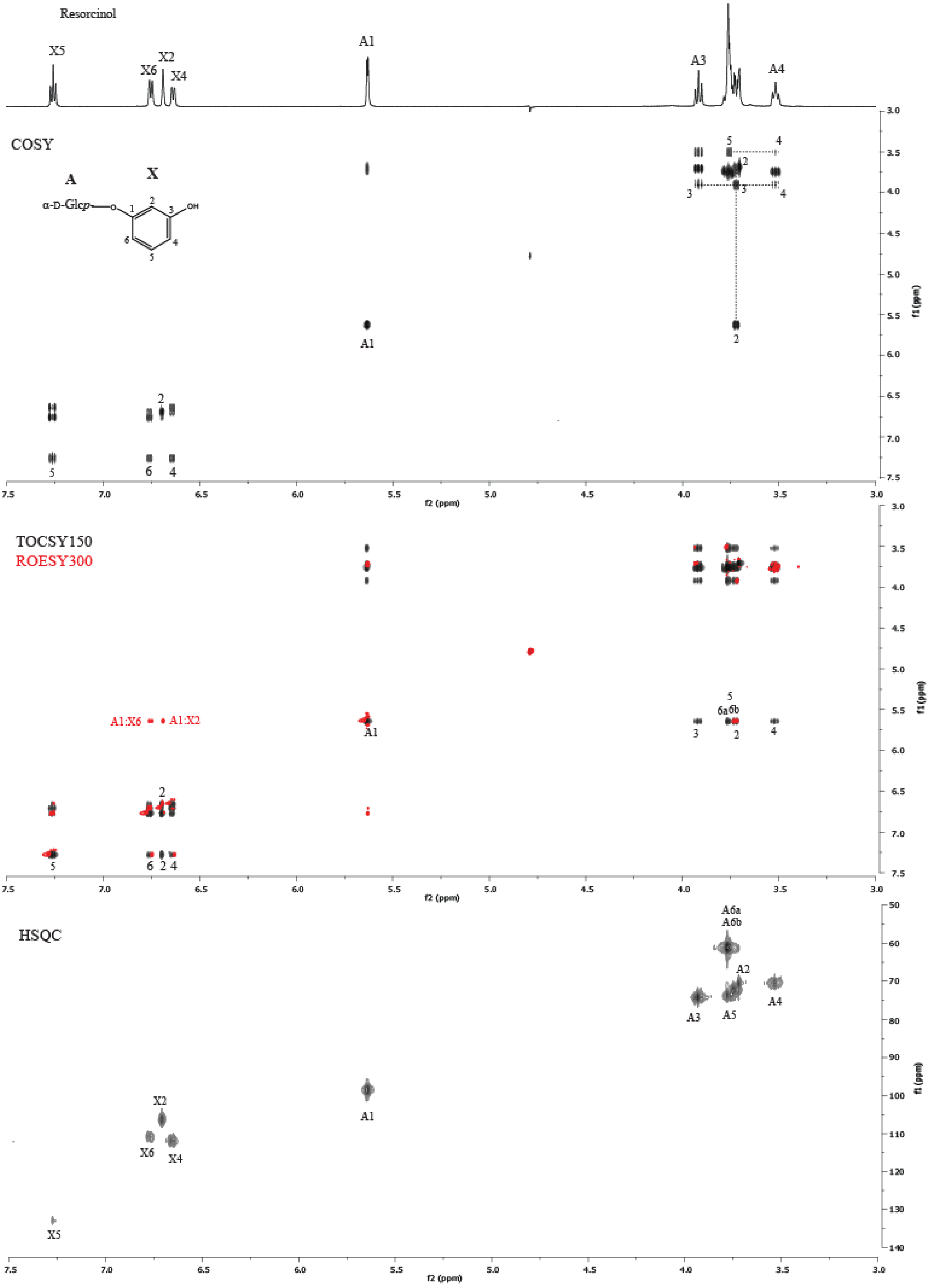

Resorcinol
X5
X6
X2
X4
A1
A3
A4
COSY
A
X
α-D-Glcp
OH
TOCSY150
ROESY300
A1:X6
A1:X2
HSQC
A6a
A6b
A2
A5
f2 (ppm)
f1 (ppm)

Figure S8

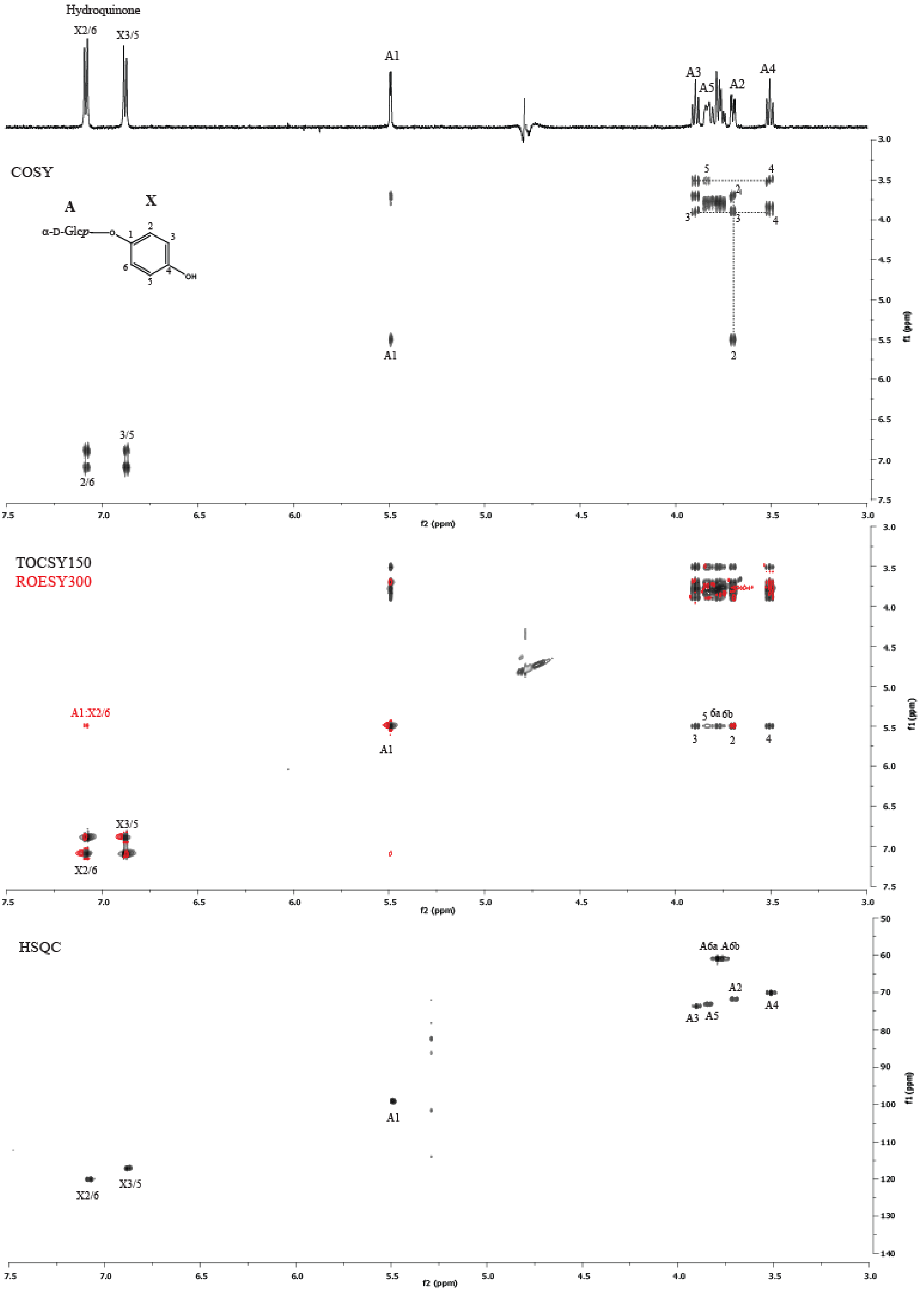
Hydroquinone
X2/6
X3/5
A1
A3
A5
A2
A4
COSY
A
X
α-D-Glcp
OH
TOCSY150
ROESY300
A1:X2/6
HSQC
A6a A6b
f2 (ppm)
f1 (ppm)

Figure S9

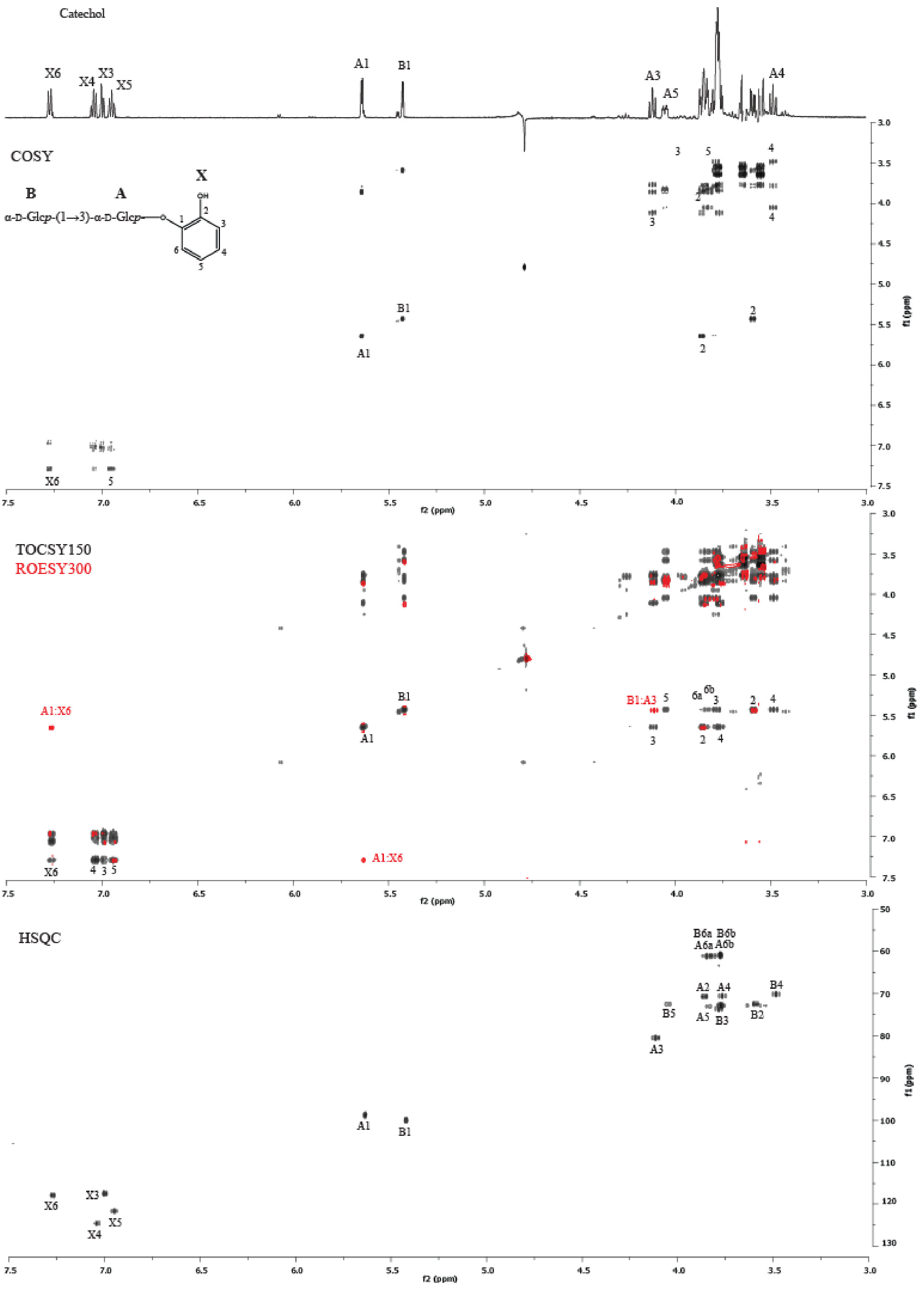
Catechol
X6
X4
X3
X5
A1
B1
A3
A5
A4
COSY
B
A
X
α-D-Glcp-(1→3)-α-D-Glcp
OH
TOCSY150
ROESY300
A1:X6
B1:A3
HSQC
B6a B6b
A6a A6b
A2 A4
B4
B5
A5 B3
B2
A3
f2 (ppm)
f1 (ppm)
